# CD8^+^ T Cells Mediate Female-Dominant IL-4 Production and Airway Inflammation in Allergic Asthma

**DOI:** 10.1371/journal.pone.0140808

**Published:** 2015-10-21

**Authors:** Chihiro Ito, Kaori Okuyama-Dobashi, Tomomitsu Miyasaka, Chiaki Masuda, Miki Sato, Tasuku Kawano, Yuichi Ohkawara, Toshiaki Kikuchi, Motoaki Takayanagi, Isao Ohno

**Affiliations:** 1 Department of Pathophysiology, Tohoku Pharmaceutical University, Miyagi, Japan; 2 Department of Respiratory Medicine and Infectious Diseases, Niigata University Graduate School of Medical and Dental Sciences, Niigata, Japan; Mie University Graduate School of Medicine, JAPAN

## Abstract

The prevalence and severity of bronchial asthma are higher in females than in males after puberty. Although antigen-specific CD8^+^ T cells play an important role in the development of asthma through their suppressive effect on cytokine production, the contribution of CD8^+^ T cells to sex differences in asthmatic responses remains unclear. In the present study, we investigated the sex-specific effect of CD8^+^ T cells in the suppression of asthma using an ovalbumin mouse model of asthma. The number of inflammatory cells in bronchoalveolar lavage (BAL) fluid, lung type 2 T-helper cytokine levels, and interleukin-4 (IL-4) production by bronchial lymph node cells were significantly higher in female wild-type (WT) mice compared with male mice, whereas no such sex differences were observed between male and female *cd8α*-disrupted mice. The adaptive transfer of male, but not female, CD8^+^ T cells reduced the number of inflammatory cells in the recovered BAL fluid of male recipient mice, while no such sex difference in the suppressive activity of CD8^+^ T cells was observed in female recipient mice. Male CD8^+^ T cells produced higher levels of IFN-γ than female CD8^+^ T cells did, and this trend was associated with reduced IL-4 production by male, but not female, CD4^+^ T cells. Interestingly, IFN-γ receptor expression on CD4^+^ T cells was significantly lower in female mice than in male mice. These results suggest that female-dominant asthmatic responses are orchestrated by the reduced production of IFN-γ by CD8^+^ T cells and the lower expression of IFN-γ receptor on CD4^+^ T cells in females compared with males.

## Introduction

Bronchial asthma is characterized by chronic airway inflammation in response to inhaled allergens, leading to wheezing, coughing, chest tightness and shortness of breath. Despite advances in both short- and long-term control medications for asthma, the incidence rate of asthma deaths is 1.4 per 10,000 persons with asthma in general population, but can be as high as 5.8 per 10,000 persons with asthma, especially in people aged 65 or over in the USA [1]. In addition, the prevalence of asthma in children aged 0–17 years has increased over the last few years [1].

Gender is an important universal risk factor for severe asthma exacerbations and death [2, 3]. Recent studies have demonstrated that the prevalence and severity of asthma in adults are higher in women than in men [4, 5]. In addition, the rate of relapse after discharge from the emergency department for acute asthma among adults is also higher in women than in men [6], which is likely associated with sex differences in resistance to controller medications such as inhaled corticosteroids [7].

In the development of asthma, allergen-specific CD4^+^ T cells play a pivotal role by facilitating airway inflammation, which is largely mediated by type 2 T-helper (Th2) cytokines such as interleukin 4 (IL-4), IL-5 and IL-13 [8]. IL-4 directly promotes the polarization of naïve CD4^+^ T cell differentiation towards Th2 phenotype, which initiates inflammatory responses and propagates the symptoms of asthma [9, 10]. Furthermore, IL-4 induces higher levels of vascular cell adhesion molecule 1 (VCAM-1) expression on endothelial cells [11], which results in the accumulation of T lymphocytes, monocytes, basophils, and, most prominently, eosinophils to the site of allergic airways. Data from *Il4*-disrupted (knockout) (IL-4KO) mice and CD4-depleted mice revealed the attenuated hallmark signs of asthma, including the accumulation of eosinophils in BAL fluid and airway hyperresponsiveness, indicating that IL-4 production from CD4^+^ T cells is a key factor in the development of asthma [12–14].

Sex-specific differences in Th2 cytokine production have been reported in both asthmatic patients and mice. Recently, Loza et al. demonstrated that greater numbers of IL-13-producing T cells differentiated from anti-CD3/CD28-stimulated peripheral blood lymphocytes taken from atopic asthmatic female subjects compared with male subjects [15]. In our previous studies using murine models of asthma, the recovery of IL-4, IL-5 and IL-13 in bronchoalveolar lavage (BAL) fluid from female mice was significantly higher than that of male mice [16]. Although female-dominant Th2 cytokine production was observed in lung and bronchial lymph node (BLN) cells [16, 17], it is not yet fully understood how these sex differences in Th2 cytokine production are generated in the development of asthma.

CD8^+^ T cells produce Th1- and Th2-associated cytokines and can function as helper T cells under Th2-polarizing conditions [18, 19]. Previous studies revealed that majority of the CD8^+^ T cells in asthma secrete IFN-γ and may act to antagonize a Th2 response [20–22]. Philippe et al. demonstrated that CD8^+^ T cells from the spleens of asthmatic mice produced large amounts of IFN-γ but little IL-4 or IL-5 [18]. The adaptive transfer of antigen-specific CD8^+^ T cells from the cervical lymph nodes increased the number of IFN-γ-producing cells in the lung, which was involved in suppressing late-phase allergic airway responses [23].

Therefore, in the present study, we aimed to determine the role that CD8^+^ T cells play in female-dominant IL-4 production in adult asthma using a mouse model. We found that CD8^+^ T cells were required for the observed sex differences in allergic airway inflammation. The sex differences in IFN-γ production by CD8^+^ T cells and IFN-γ receptor expression on CD4^+^ T cells may be a necessary and sufficient for female-dominant IL-4 production in asthma.

## Materials and Methods

### Mice

Male and female wild-type (WT) C57BL/6 mice and *cd8α*-disrupted (knockout) CD8KO mice were purchased from CLEA Japan (Osaka, Japan) and the Jackson Laboratory (Bar Harbor, ME, USA), respectively. All mice were kept under specific pathogen-free conditions at the Institute for Animal Experimentation, Tohoku Pharmaceutical University (Sendai, Japan). All experimental procedures involving animals were approved by the Committee of Animal Experiments at Tohoku Pharmaceutical University (approved numbers: 12023-cn, 13001-cn-a and 14002-cn). We took the utmost care to alleviate any pain and suffering on the part of the mice.

### Sensitization and antigen challenge

Mice at 6 to 8 weeks of age were sensitized by intraperitoneal injections of 8 μg of ovalbumin (OVA; Grade V, Sigma-Aldrich, St Louis, MO, USA) adsorbed with aluminum hydroxide (Wako Pure Chemical Industries, Osaka, Japan) on days 0 and 5 [24]. On day 30, the mice were challenged with aerosolized OVA (0.5% in saline) for 1 h on two occasions 4 h apart.

### Preparation of bronchoalveolar lavage fluids

BAL samples were collected with 2 x 0.25 ml of chilled PBS through a cannula inserted in the trachea. Total cell numbers recovered from the BAL fluid were counted with a hemocytometer. Then, 2 x 10^5^ cells were centrifuged onto a glass slide using a Cytospin 4 (Shandon, Runcorn, UK) and then stained with Diff-Quick solution (International Reagents Corp., Kobe, Japan). Cell differential percentages were determined by counting a minimum of 200 cells under light microscopy.

### Preparation of lung homogenates

The lung were excised one day after OVA challenge from WT and CD8KO mice and homogenized in chilled 0.1% Triton-X PBS with 1% protease inhibitor (Sigma-Aldrich). After centrifugation at 15,000 x g for 15 min at 4°C, the supernatants were stored at −80°C for subsequent cytokine assays.

### Cell preparation and stimulation

BLN cells were prepared from WT and CD8KO mice (5 to 14 mice for one experiment) one day after OVA challenge as previously described [17]. BLN cells (4 x 10^5^ cells/well) were cultured in the presence of OVA for 3 days. Lipopolysaccharide (LPS) prepared from *Escherichia coli* O-111 (Sigma-Aldrich) was used as a control. CD4^+^ T cells and CD8^+^ T cells in BLN, and CD11c^+^ cells in spleen of sensitized and challenged male and female WT mice were purified by positive selection on an autoMACS Separator (Miltenyi Biotec, Bergish Gladbach, Germany) using anti-mouse CD4 (L3T4), anti-mouse CD8α (Ly-2) and anti-mouse CD11c MicroBeads (Miltenyi Biotec), respectively. CD4^+^ T cells were co-cultured with male or female CD8^+^ T cells and CD11c^+^ cells in the presence of OVA for 3 days. CD11c^+^ cells were prepared as a mixture of male and female CD11c^+^ cells at ratio of 1:1. Because we previously reported the functional difference in CD11c^+^ cells from BLN between male and female mice [25], it is possible that the function of splenic CD11c^+^ cells were different between the sexes. Therefore, in order to exclude the influence of sex differences in the antigen presenting cells, the mixture of CD11c^+^ cells was used in this study. A 10 μg/ml of anti-IFN-γ neutralizing antibody (Ab) (PeproTech, Inc., Rocky Hill, New Jersey, USA) was used to block the effects of IFN-γ. In another experiment, CD4^+^ T cells and CD11c^+^ cells were cultured with 10 ng/ml of recombinant (r)IFN-γ (PeproTech) [26]. Naïve CD4^+^ T cells were isolated from spleen of male and female WT mice using autoMACS Separator and cultured with rIFN-γ (10 ng/ml) for 72 h. The concentration of IFN-γ (10 ng/ml) [26] used in the current study was about 1,000 times higher than that, the levels of pg/ml, in BAL fluids and lung homogenates in mice models of allergic asthma [27]. However, the concentration at the site of inflammation would be higher than the concentration in samples such as BAL fluids and lung homogenates in consideration of the sampling procedures.

### Flow cytometric analysis

The BAL cells were preincubated with anti-FcRII and III mAb on ice for 15 min in PBS containing 1% fetal calf serum (FCS) and 0.1% sodium azide, and stained with APC-conjugated anti-CD3 (Clone 145-2C11; BioLegend, San Diego, CA, USA), FITC-conjugated anti-CD4 (clone GK1.5; BioLegend) and peridinin-chlorophyll protein complex (PerCP)-conjugated anti-CD8 (Clone 53–6.7; BioLegend). For intracellular staining of IL-4 and IFN-γ on T cells, BLN cells were isolated from WT mice one day after challenge, and cultured at 2 x 10^5^ cells with 5 ng/ml of phorbol 12-myristate 13-acetate, 500 ng/ml of ionomycin and 2 μM of monensin (Sigma-Aldrich) for 4 hours at 37°C before the cell surface was stained. Then, Fc receptors on cell surface were blocked, and cells were stained with PerCP-conjugated anti-CD3 (Clone 145-2C11; BioLegend) and FITC-conjugated anti-CD4 (BioLegend) or FITC-conjugated anti-CD8 mAbs (clone 53–6.7; BD Biosciences Pharmingen, San Diego, CA, USA). The isotype-matched control IgG for each Ab was used as a reference. Cells were then incubated in the presence of cytofix/cytoperm (BD Biosciences Pharmingen), washed twice in BD perm/wash solution and stained with phycoerythrin (PE)-conjugated anti-IL-4 (clone 11B11; Biolegend), PE-conjugated anti-IFN-γ mAbs (clone XMG1.2; eBiosciences) or control rat IgG. For evaluation of IFN-γ receptor expression on CD4^+^ T cells, BLN cells obtained from asthmatic WT mice and splenic CD4^+^ T cells from naïve WT mice were stained with PE-conjugated anti-mouse CD119 (anti-IFN-γ receptor α, clone 2E2; eBioscience) or anti-IFN-γ receptor β chain (clone MOB-47; BioLegend). The stained cells were analyzed using a BD FACSCalibur flow cytometer (BD Biosciences, San Jose, CA, USA) or BDAriaII flow cytometer (BD Biosciences). Data were collected from 30,000 individual cells using parameters of forward scatter (FSC) and side scatter (SSC) to limit the lymphocyte population. The number of CD4^+^ and CD8^+^ T cells was estimated by multiplying the lymphocyte number, calculated as mentioned above, by the proportion of each subset.

### Quantitative real-time RT-PCR analysis

Total RNA was extracted from the CD8^+^ T cells using an RNeasy Micro Kit (QIAGEN, Valencia, CA, USA), and the first-strand cDNA was synthesized using PrimeScript RT reagent kit with gDNA Eraser (TaKaRa Bio Inc., Otsu, Japan). Quantitative real-time reverse transcription (RT)-PCR was performed using gene-specific primer and Power SYBR Green PCR Master Mix (Applied Biosystems, Foster City, CA, USA) in a StepOnePlus Real-Time PCR System (Applied Biosystems). The primer sequences used for amplification are shown in Table 1. The expression levels of target genes and hypoxanthine-guanine phosphoribosyltransferase (*Hprt*) as a reference were calculated for each sample using the reaction efficiency determined using standard amplifications.

**Table 1:** Primer sequences for quantitative real-time RT-PCR.

	Forward (5'-3')	Reverse (5'-3')
IFN-γ	ATCTGGAGGAACTGGCAAAAGGATG	ATGACGCTTATGTTGTTGCTGATGG
T-bet	CACTAAGCAAGGACGGCGAATG	GTCCACCAAGACCACATCCACAA
GATA-3	TCTGGAGGAGGAACGCTAATGGG	TTCGGGTCTGGATGCCTTCTTTC
IL-10	GCACTACCAAAGCCACAAGGCA	CAGTAAGAGCAGGCAGCATAGCA
TGF-β_1_	TGATACGCCTGAGTGGCTGTC	CCTGTATTCCGTCTCCTTGGTTCA
Perforin	ACTACGGCTGGGATGATGACCTT	GTGAGATGGGGCAGACACTTGG
Fas ligand	TTCTGGTGGCTCTGGTTGGAATG	ACTGGGGTTGGCTATTTGCTTTTC
HPRT	TTGGGCTTACCTCACTGCTTTCC	ATCATCGCTAATCACGACGCTGG

HPRT: hypoxanthine-guanine phosphoribosyltransferase.

### Measurement of cytokine concentration

The levels of IL-2, IL-4 and IFN-γ in lung homogenates and culture supernatants were assayed using an ELISA kits (eBioscience, San Diego, CA, USA). The detection limits were 2 pg/ml for IL-2, 4 pg/ml for IL-4 and 15 pg/ml for IFN-γ. The contents of IL-5 and IL-13 in the lung homogenates were measured using an ELISA kits (R&D systems, Minneapolis, MN, USA). The limits of detection were 7 and 1.5 pg/ml, respectively. Total protein levels of lung homogenates were measured using a detergent-compatible protein assay kit (Bio-Rad Laboratory, Hercules, CA, USA). The cytokine concentration was adjusted for the protein level of each lung.

### Adaptive transfer of CD8^+^ T cells

CD8^+^ T cells (4 x 10^5^ cells/mouse) were transferred into OVA-sensitized CD8KO mice via the tail vein. Three days later, mice were challenged with aerosolized OVA.

### Statistical analysis

Statistical analysis was conducted using GraphPad Prism 5 software (GraphPad Software, La Jolla. CA, USA). Differences between the two groups were tested using two-tail analysis in an unpaired Student’s *t*-test. Differences among three groups or more were tested using ANOVA with post hoc analysis (Student-Newman-Keuls test). A *p*-value of less than 0.05 was considered significant.

## Results

### Involvement of CD8^+^ T cells in sex differences in asthma features

**Fig 1:**
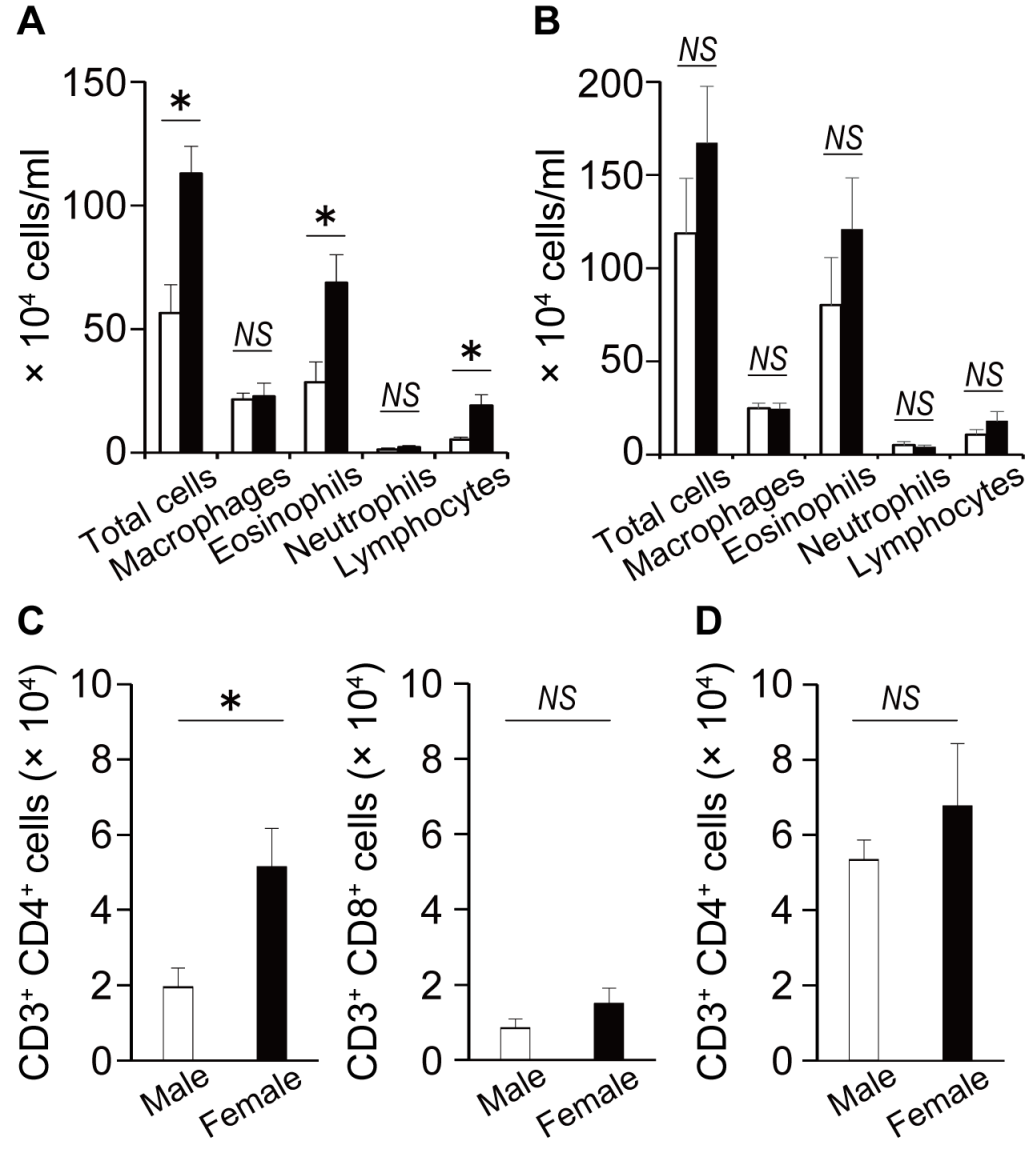
Sex differences in inflammatory cell infiltration in BAL fluid. Male and female WT mice (A) and CD8KO mice (B) were sensitized and challenged. The number of inflammatory cells in BAL fluids were counted on day 5 after OVA challenge. Data are shown as the mean ± SEM from at least three independent experiments (n = 12–16). (C and D) The number of CD4^+^ and CD8^+^ T cells in BAL fluids of WT mice (C) and CD8KO mice (D) on day 5 after OVA challenge is shown. Each column represents the mean ± SEM of four to five mice. Open bars, male mice; closed bars, female mice.*, P < 0.05; NS, not significant.

**Fig 2:**
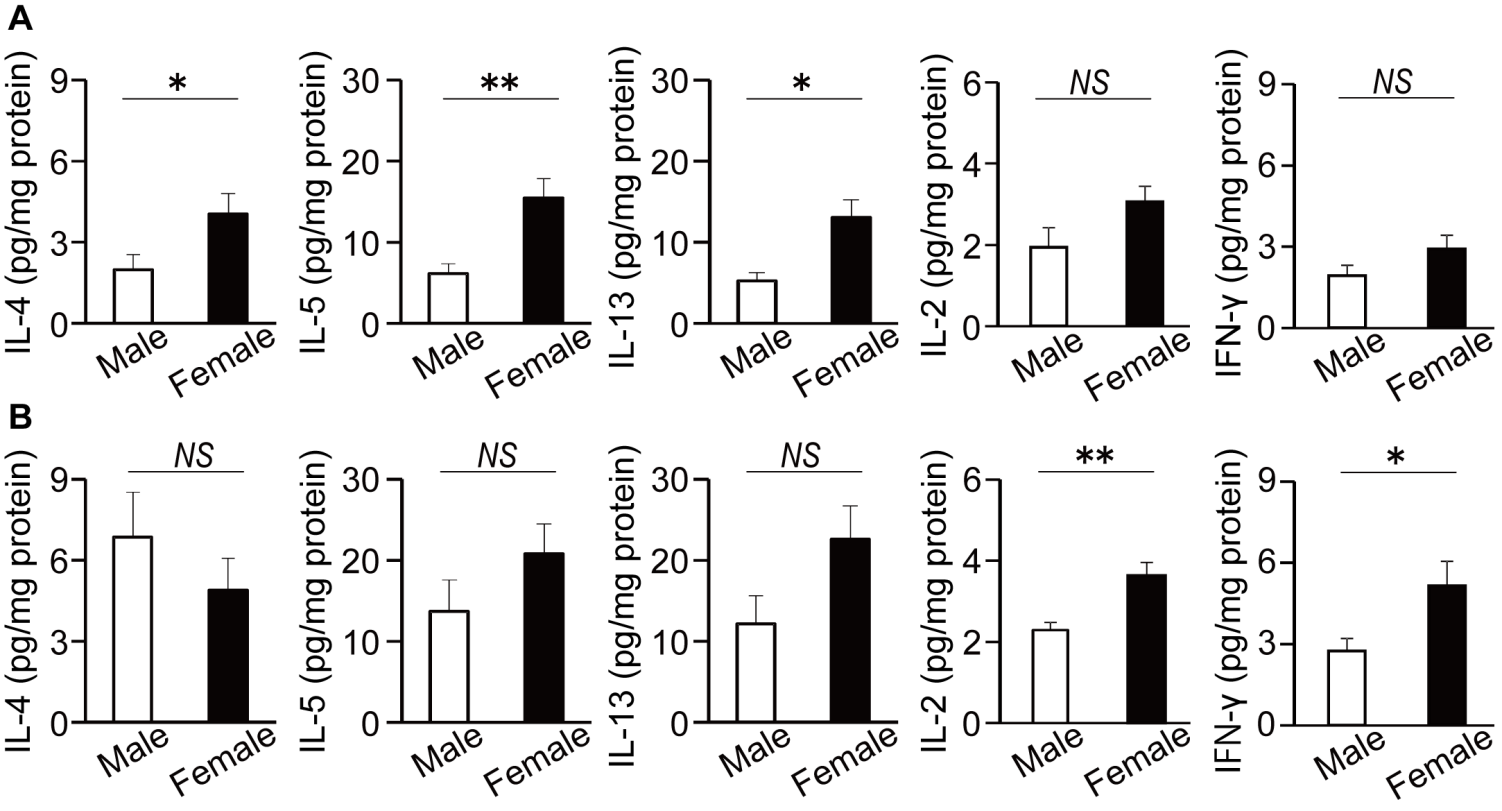
Sex differences in cytokine production in lung. Male and female WT mice (A) and CD8KO mice (B) were sensitized and challenged. IL-2, IL-4, IL-5, IL-13 and IFN-γ levels in the lung were measured by ELISA one day after OVA challenge. Data are shown as the mean ± SEM from at least three independent experiments (n = 8–11). Open bars, male mice; closed bars, female mice. *, P < 0.05; **, P < 0.01; NS, not significant.

To clarify the sex differences in prototypical asthma-related features in WT mice, we initially compared the development of airway inflammation between male and female WT mice. As shown in Fig 1A, the numbers of total cells, eosinophils and lymphocytes in the BAL fluid of female WT mice were significantly higher than those in male WT mice. Furthermore, the number of CD4^+^ T cells in BAL fluids of WT mice was significantly higher in female mice than in male mice, whereas CD8^+^ T cell counts in BAL fluids were not different between male and female mice (Fig 1C). By contrast, the sex differences in differential cell counts and the number of CD4^+^ T cells in BAL fluids were abrogated in CD8KO mice (Fig 1B and 1D). Furthermore, the production of Th2 cytokines such as IL-4, IL-5 and IL-13 in the lung were significantly increased in female WT mice compared to male WT mice (Fig 2A), although there was no apparent difference between male and female CD8KO mice (Fig 2B). In the case of Th1 cytokine production, the sex differences in IL-2 and IFN-γ contents in the lung were not observed between male and female WT mice, whereas those cytokines were significantly higher in female CD8KO mice than male CD8KO mice (Fig 2A and 2B). These data indicate that CD8^+^ T cells may be involved in the development of the sex differences in asthmatic responses, including Th1 and Th2 cytokine production, and eosinophil and lymphocyte infiltration in the lungs.

### Involvement of CD8^+^ T cells in female-dominant IL-4 production by bronchial lymph node cells

**Fig 3:**
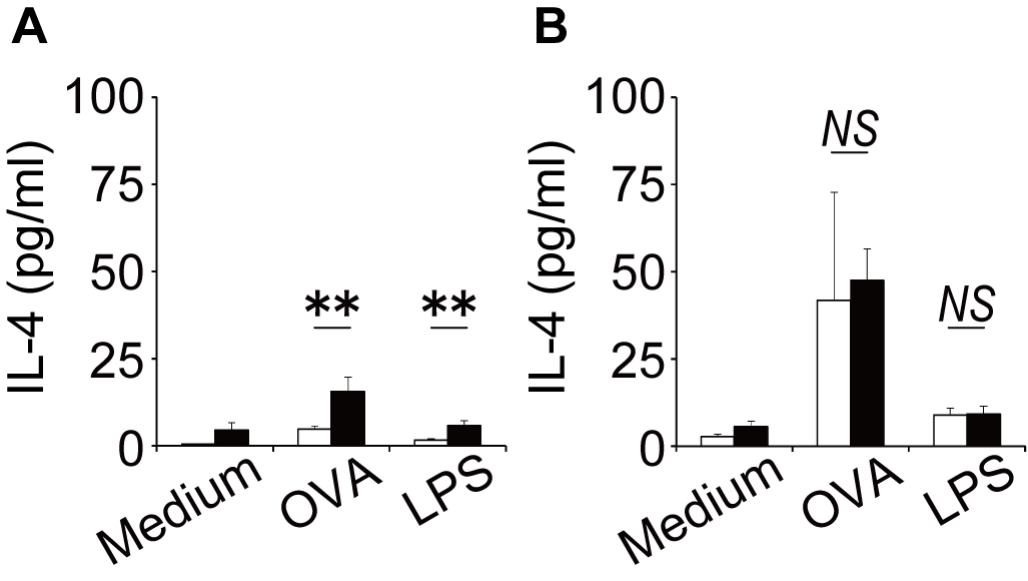
Sex differences in IL-4 production from BLN cells. BLN cells from WT mice (A) and CD8KO (B) mice were prepared one day after OVA inhalation and cultured with 3 μg/ml of OVA or 1 μg/ml of LPS for 72 h. The concentration of IL-4 in the culture supernatants was measured by ELISA. Data are shown as the mean ± SD of triplicate cultures. Experiment were repeated twice with similar results. Open bars, BLN cells from male mice; closed bars, BLN cells from female mice. **, P < 0.01; NS, not significant.

The pathogenesis of asthma is directed by allergen-specific Th2 cells generated in the secondary lymphoid organs such as the BLN, and the migration of Th2 cells into lung plays an important role in mobilization of eosinophils via the secretion of IL-4 and IL-5 [28]. Therefore, to address the role of CD8^+^ T cells in sex differences in cellular responses to inhaled allergen, we examined how the lack of CD8^+^ T cells affected the production of IL-4 by BLN cells upon stimulation with OVA. As shown in Fig 3A, the production of IL-4 by BLN cells upon stimulation with OVA was significantly higher in female WT mice than in males. A similar pattern was observed when T cells were non-specifically activated via LPS. In contrast, female-dominant IL-4 production by BLN cells upon stimulation was not observed in CD8KO mice (Fig 3B).

### Male CD8^+^ T cells show reduced IL-4 production by male CD4^+^ T cells

**Fig 4:**
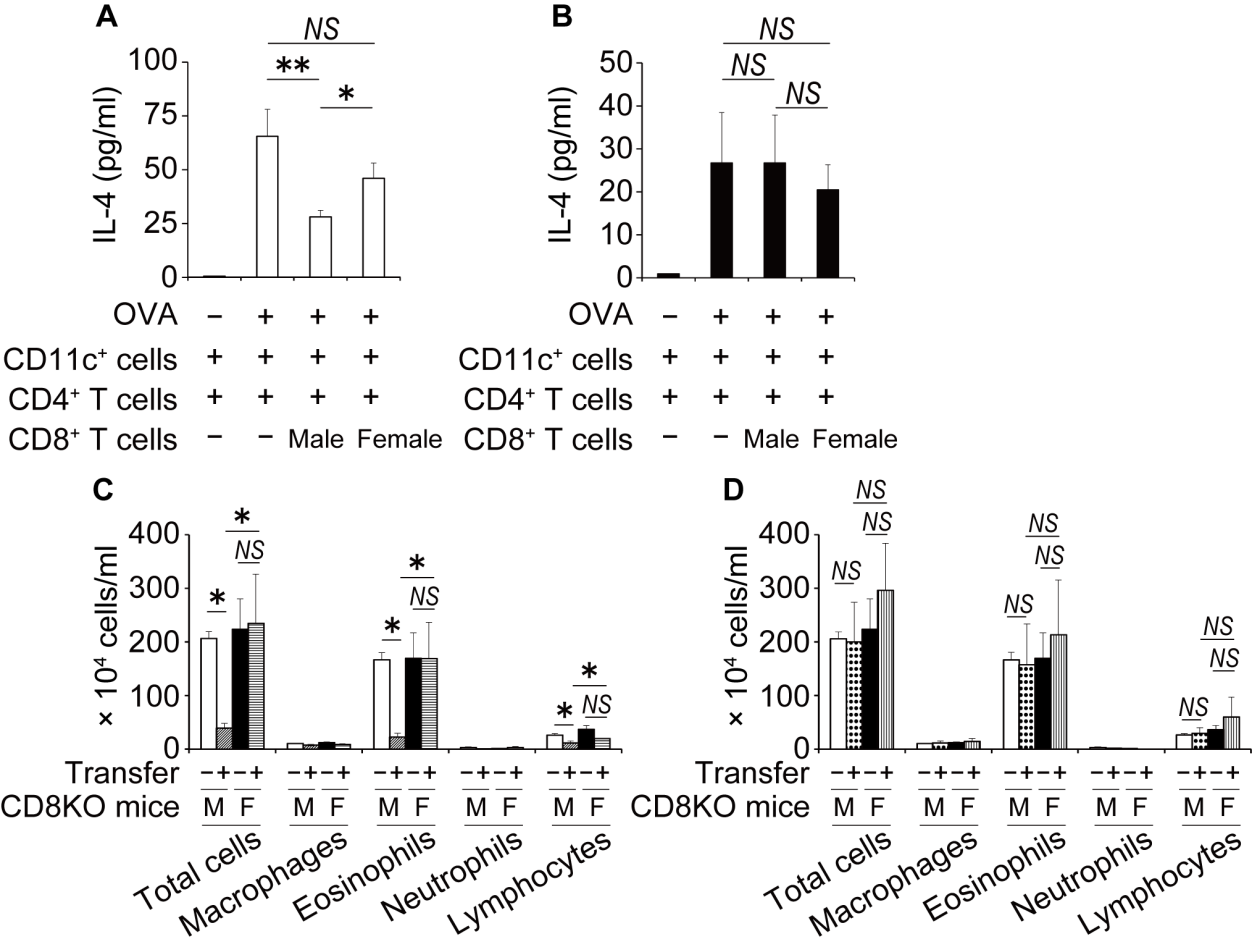
Sex differences in the inhibitory effect of BLN CD8^+^ T cells. Male (A) or female (B) CD4^+^ T cells (2 x 10^5^ cells/well) were cultured with CD8^+^ T cells (2 x 10^5^ cells/well) and splenic CD11c^+^ cells (2 x 10^5^ cells/well) in the presence of OVA (50 μg/ml) for 72 h. Concentration of IL-4 in the culture supernatants was measured by ELISA. Data are shown as the mean ± SD of triplicate cultures. Experiment were repeated twice with similar results. (C and D) Sensitized male or female CD8KO mice were adoptively transferred with CD8^+^ T cells from BLN of sensitized and challenged male (C) or female (D) WT mice. Saline was used as control of the transfer. Three days later, recipient CD8KO mice were challenged with OVA aerosol, and then sacrificed on day 5 post-OVA challenge. The numbers of inflammatory cells in BAL fluids were counted. Data are shown as the mean ± SEM from two independent experiments (n = 4–9). Open bars, male mice transferred with saline; closed bars, female mice transferred with saline; hatched bars, male mice transferred with male CD8^+^ T cells; horizontal line bars, female mice transferred with male CD8^+^ T cells; dotted bars, male mice transferred with female CD8^+^ T cells; vertical line bars, female mice transferred with female CD8^+^ T cells. −, mice transferred with saline; +, mice transferred with CD8^+^ T cells; *, P < 0.05; **, P < 0.01; NS, not significant.

CD8^+^ T cells attenuate allergic inflammation and play a central role in moderating Th2 polarization within the lymph nodes during allergic sensitization [29]. Therefore, to address the sex difference in suppressive effects of CD8^+^ T cells on IL-4 production by CD4^+^ T cells, male or female CD4^+^ T cells derived from BLN were cultured with male or female CD8^+^ T cells isolated from BLN cells of WT mice in the presence of CD11c^+^ cells as antigen-presenting cells with OVA. As shown in Fig 4A, the amount of IL-4 in the cultures of male CD4^+^ T cells were significantly reduced in the presence of male CD8^+^ T cells, but not in the presence of female CD8^+^ T cells, compared with the amount recovered in the absence of CD8^+^ T cells. In contrast, the amount of IL-4 in the cultures of female CD4^+^ T cells was not reduced in the presence of either male or female CD8^+^ T cells, compared with the amount in the absence of CD8^+^ T cells (Fig 4B). These results suggest that the attenuation of CD8^+^ T cell-mediated suppression may play a role in the elevated IL-4 production in the BLN of female mice.

### Male CD8^+^ T cells contribute to the suppression of inflammatory cell infiltration in the BAL fluid of male mice

We next examined the functional relevance of the suppressive effect of CD8^+^ T cells on the infiltration of inflammatory cells into the lung. BLN CD8^+^ T cells from male or female WT mice were transferred intravenously into sensitized CD8KO mice that then inhaled OVA on day 3 after the cell transfer. As shown in Fig 4C, the transfer of male CD8^+^ T cells into male CD8KO mice significantly reduced the numbers of total cells, eosinophils and lymphocytes in BAL fluids compared with saline transfer. The transfer of male CD8^+^ T cells into female CD8KO mice, however, did not have this effect. In contrast, the transfer of female CD8^+^ T cells to neither male nor female CD8KO mice significantly reduced the numbers of inflammatory cells in BAL fluid compared with saline transfer (Fig 4D).

### The involvement of IFN-γ in the suppressive effect of male CD8^+^ T cells

**Fig 5:**
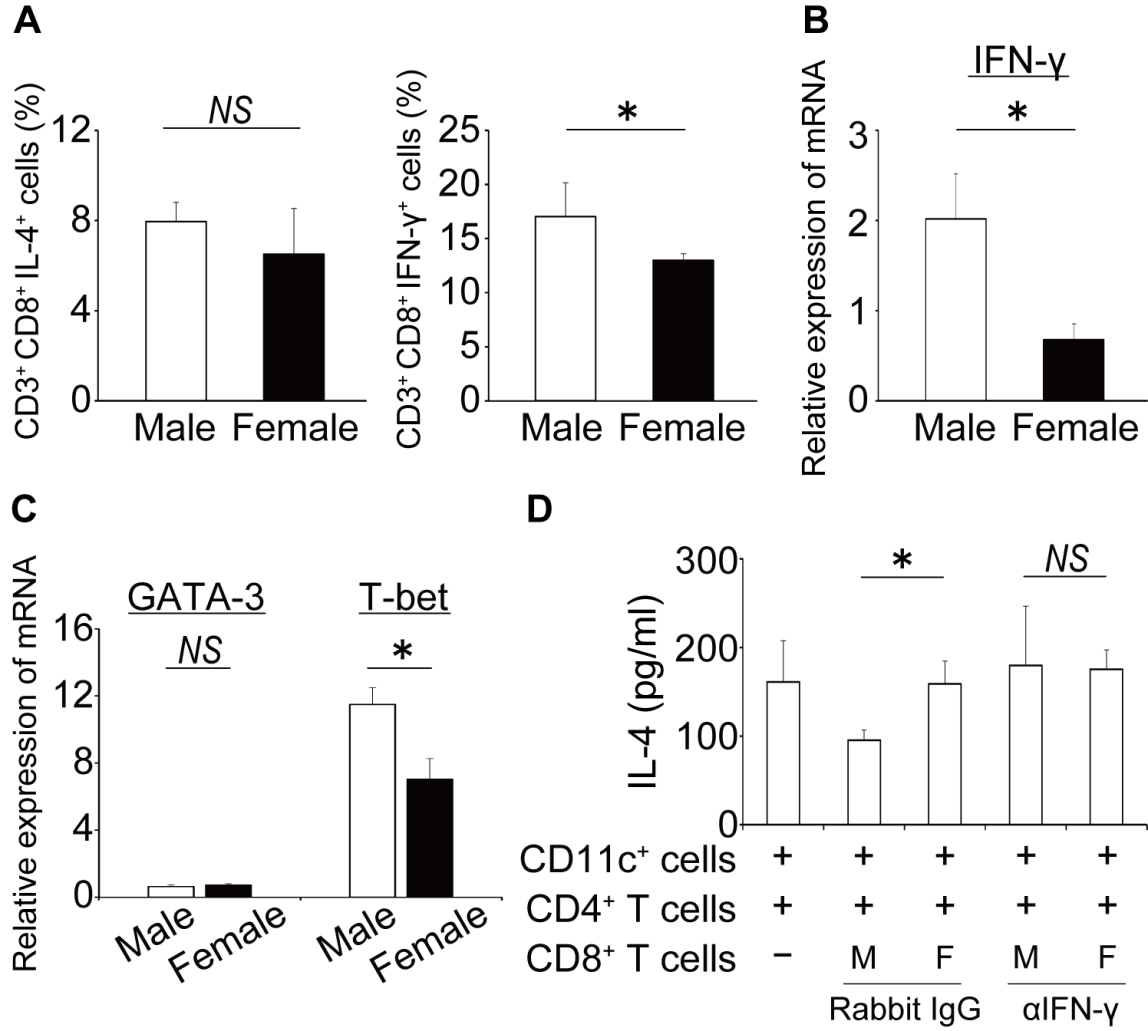
Involvement of IFN-γ in sex differences in the inhibitory effect of BLN CD8^+^ T cells. (A) Intracellular cytokine expression by CD8^+^ T cells was analyzed using a flow cytometer. Data are shown as the mean ± SD of four mice. The expression of *Ifng* (B), *Gata3* and *Tbet* (C) in CD8^+^ T cells were measured by quantitative real-time RT-PCR. Data are shown as the mean ± SEM of four to eight mice. (D) Male CD4^+^ T cells (2 x 10^5^ cells/well), CD11c^+^ cells (2 x 10^5^ cells/well) and CD8^+^ T cells (0.66 x 10^5^ cells/well) were cultured with 50 μg/ml of OVA in presence of an anti- IFN-γ mAb (10 μg/ml) or control IgG for 72 h. The concentration of IL-4 in the culture supernatants was measured by ELISA. Data are shown as the mean ± SD of triplicate cultures. Experiment were repeated twice with similar results. *, P < 0.05; NS, not significant.

It has been established that IFN-γ plays an important role in suppressing the development of Th2 cells as well as Th2 cytokine production [30]. Therefore, we first compared the expression of IFN-γ in male and female CD8^+^ T cells isolated from BLN one day after OVA challenge. As shown in Fig 5A, IFN-γ production in CD8^+^ T cells was significantly higher in male WT mice than female WT mice, although IL-4 production did not differ between male and female CD8^+^ T cells. In line with these data, the expression of *Ifng* mRNA in CD8^+^ T cells was also higher in male WT mice than in female WT mice (Fig 5B). These results suggest that the induction of type-1-cytokine-producing CD8^+^ T cell responses may be facilitated in the BLN of male WT mice under allergic asthma conditions. This possibility was supported by the finding that *Tbet* expression levels were significantly higher in male CD8^+^ T cells than in female CD8^+^ T cells, whereas *Gata3* expression levels did not differ between male and female CD8^+^ T cells (Fig 5C). In addition, we sought to establish whether the addition of an anti-IFN-γ monoclonal (m)Ab would alter the observed sex difference in the suppressive effect of CD8^+^ T cells on IL-4 production by male CD4^+^ T cells. As shown in Fig 5D, this treatment completely abrogated the sex difference in the suppressive activity of CD8^+^ T cells on IL-4 production from male CD4^+^ T cells, whereas the addition of control rat IgG did not.

### The expression of the IFN-γ receptor on CD4^+^ T cells is implicated in the sex differences in CD8^+^ T cell-mediated suppression

**Fig 6:**
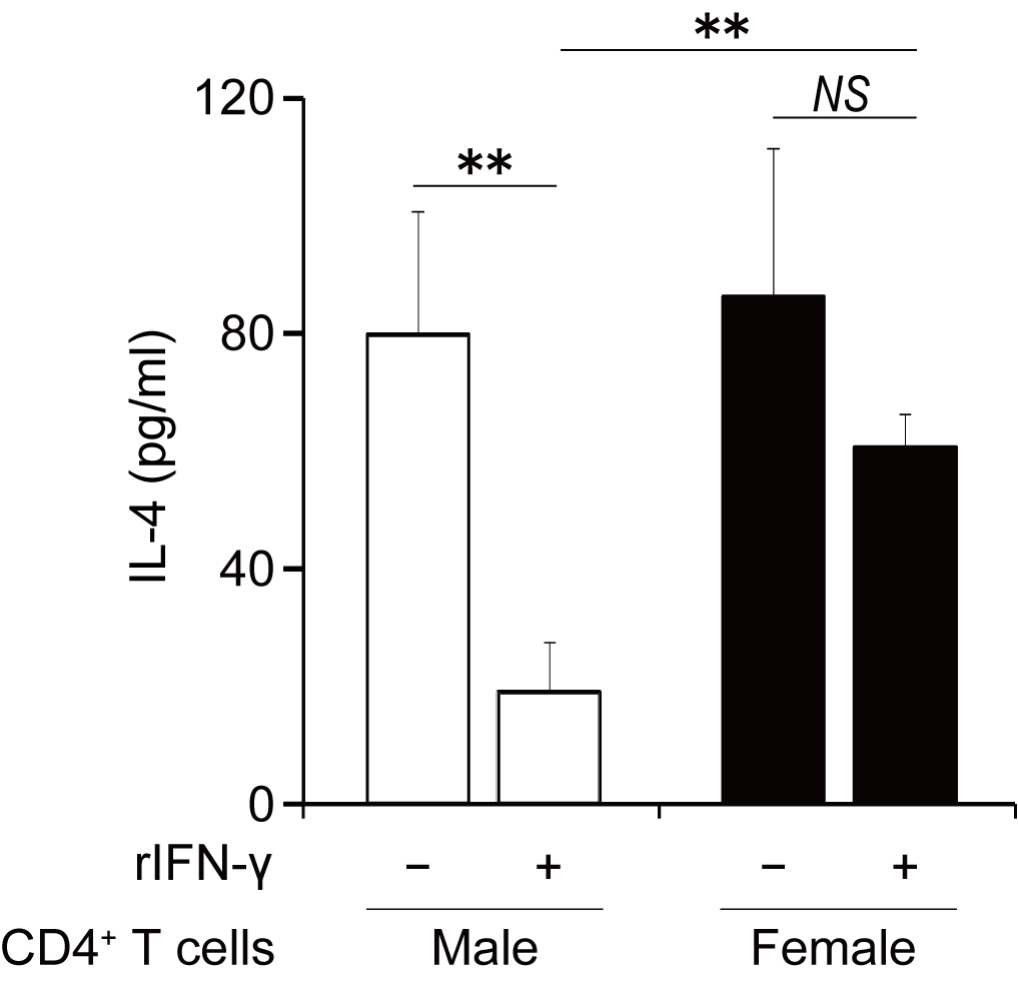
IFN-γ attenuates IL-4 production from BLN male CD4^+^ T cells. Male or female CD4^+^ T cells (2 x 10^5^ cells/well) and CD11c^+^ cells (2 x 10^5^ cells/well) were cultured with 50 μg/ml of OVA in presence of rIFN-γ (10 ng/ml) or vehicle for 72 h. Data are shown as the mean ± SD of triplicate cultures. Experiments were repeated twice with similar results. Open bars, male CD4^+^ T cells; closed bars, female CD4^+^ T cells. **, P < 0.01; NS, not significant.

**Fig 7:**
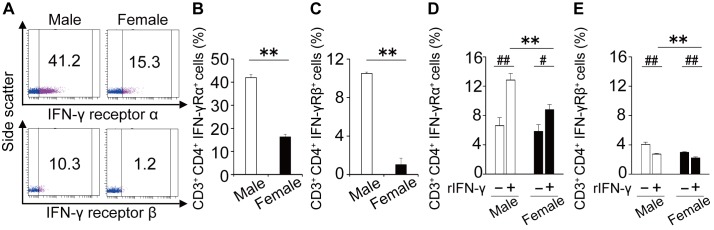
Sex differences in IFN-γ receptor expression on CD4^+^ T cells from WT mice. (A) Representative profiles of IFN-γ receptor expression on CD4^+^ T cells in BLN of male and female mice are shown. Cut-off lines were determined on the basis of an IgG isotype-matched control profile. The percentages of IFN-γ receptor α^+^ population (B) and IFN-γ receptor β^+^ population (C) in CD4^+^ T cells were analyzed in each group. Data are shown as the mean ± SD of three to five mice. Experiment were repeated twice with similar results. (D and E) CD4^+^ T cells obtained from naïve WT mice were cultured with rIFN-γ (10 ng/ml) for 72 h. The percentage of IFN-γ receptor α^+^ population (D) and IFN-γ receptor β^+^ population (E) in CD4^+^ T cells were analyzed before and after the culture. Data are shown as the mean ± SD of triplicate cultures. Experiments were repeated twice with similar results. **, P < 0.01 compared with male mice; #, P < 0.05; ##, P < 0.01 compared to vehicle pretreatments.

In Fig 4, the suppressive effects of male CD8^+^ T cells on IL-4 production were observed in the cultures of male CD4^+^ T cells but not female CD4^+^ T cells. Furthermore, those effects of male CD8^+^ T cells on the accumulation of inflammatory cells into the airway were observed in CD8KO male, but not female, mice. These results suggest that the sex difference in asthmatic responses may be due to both sex-specific functional differences in CD8^+^ T cells in terms of IFN-γ production and CD4^+^ T cells in terms of the sensitivity to suppressive stimuli such as IFN-γ. To address this possibility, we examined sex differences in the susceptibility of CD4^+^ T cells to the inhibitory activity of CD8^+^ T cells. As shown in Fig 6, IL-4 production from male CD4^+^ T cells was reduced by adding rIFN-γ, whereas this effect was not observed in female CD4^+^ T cells. Furthermore, the amount of IL-4 produced by female CD4^+^ T cells was significantly higher than male CD4^+^ T cells in the presence of IFN-γ, whereas the amount of IL-4 produced by CD4^+^ T cells did not differ between male and female mice in the absence of IFN-γ. These results suggest that sex differences in the susceptibility of CD4^+^ T cells to IFN-γ are implicated in the sex differences in IL-4 production by CD4^+^ T cells. In agreement with these data, the expression of the IFN-γ receptor α (Fig 7A and 7B) and β chain (Fig 7A and 7C) on CD4^+^ T cells was strikingly lower in female mice compared with male mice.

### The involvement of IFN-γ in the sex differences in IFN-γ receptor expression on CD4^+^ T cells

To further address the mechanisms that regulate sex differences in the expression of IFN-γ receptors on CD4^+^ T cells, we examined the effect of IFN-γ on IFN-γ receptor α and β expression on WT naïve CD4^+^ T cells. As shown in Fig 7D and 7E, the proportion of IFN-γ receptor α^+^ CD4^+^ T cells was significantly increased in both male and female mice, although the proportion of IFN-γ receptor β^+^ CD4^+^ T cells was significantly reduced in both male and female mice. In addition, there was a significant difference in the proportion of IFN-γ receptor α^+^ CD4^+^ and receptor β^+^ CD4^+^ T cells between male and female mice after the treatment.

## Discussion

This is the first report demonstrating that CD8^+^ T cells play an important role in the development of sex difference in allergic airway inflammation, indicated by the following findings that i) the number of eosinophils, lymphocytes and CD4^+^ cells in BAL fluid (Fig 1A and 1C) and the levels of recovered Th2 cytokines in the lung (Fig 2A) were greater in female WT mice than male WT mice; ii) this female-dominant allergic inflammation was not observed in CD8KO mice (Figs 1B, 1D and 2B); and iii) the adoptive transfer of male CD8^+^ T cells significantly reduced the infiltration of inflammatory cells in the BAL fluid of male but not female CD8KO mice leading to female-dominant airway inflammation, whereas that of female CD8^+^ T cells did not (Fig 4C and 4D). By comparison of WT mice with CD8KO mice in each sex, the sex differences in allergic airway inflammation were likely to depend on higher suppression by CD8^+^ T cells in male than female mice, confirmed by the CD8^+^ T cell transfer experiments. The inhibitory role of CD8^+^ T cells in the regulation of allergic inflammatory responses has been extensively investigated, although the sex-related function of CD8^+^ T cells in the regulation of allergic asthma has never been investigated. Tang and coworkers reported that antigen-specific effector CD8^+^ T cells attenuated allergic pulmonary inflammation by enhancing IFN-γ production and altering the ability of pulmonary dendritic cells (DCs) to polarize OVA-specific CD4^+^ T cells toward a Th1 phenotype [31]. The depletion of CD8^+^ T cells increased the circulating levels of antigen-specific IgE and IgG1, and airway reactivity and remodeling [18, 32]. Wells et al. reported that the transfer of CD8^+^ T cells from OT-I transgenic mice resulted in a marked suppression of airway eosinophilia and Th2 lung cytokine responses at least partly through the stimulation of IL-12 in the lung, reflecting their bias toward the cytotoxic type 1 (Tc1) phenotype [33].

We previously demonstrated the involvement of immune cells in the sex-related allergic airway inflammation in the study of the adoptive transfer of splenocytes from sensitized mice to naïve mice in which the most severe airway inflammation was observed in female mice transferred with female splenocytes [16]. The increased number of macrophages in airways of female mice has been reported to contribute to the sex difference in allergic airway inflammation in mice [34]. Furthermore, we have reported differential function of CD11c^+^ cells from BLN in the activation of CD4^+^ cells [25]. Accordingly, various types of cells involved in Th2 immune responses such as dendritic cells and NKT cells would be promising targets in investing mechanisms for the sex difference in asthma pathophysiology.

CD8^+^ T cells could contribute to the development of the sex differences mainly by the induction of female-dominant Th2 differentiation, suggested by current findings that IL-4 production by BLN cells stimulated with OVA was greater in female WT mice than male WT mice (Fig 3A), whereas this female-dominant IL-4 production was not observed in CD8KO mice (Fig 3B). Female-dominant Th2 differentiation is supported by our previous findings that Th2 cells identified as T_1_/ST_2_^+^ CD4^+^ T cells were increased in female BLN cells stimulated with OVA compared to male BLN cells [17]. Interestingly, IL-4 production by CD4^+^ T cells either after the culture with OVA and APCs (Fig 6) or after the stimulation with PMA/Ionomycin (S1 Fig) was not different between the sexes. However, in the case of male CD4^+^ T cells, the presence of male, but not female, CD8^+^ T cells decreased IL-4 production after the culture with OVA and APCs (Fig 4A), while neither male nor female CD8^+^ T cells did in the case of female CD4^+^ T cells (Fig 4B). These findings suggest that male-dominant Th2-suppression by CD8^+^ T cells might result in female-dominant Th2 differentiation, leading to higher suppression of allergic airway inflammation by CD8^+^ T cells in male than female mice as discussed above.

As one of underlying mechanisms for male-dominant Th2-suppression by CD8^+^ T cells, the interaction of IFN-γ with its receptors was suggested by the following findings that i) the production of IFN-γ from male CD8^+^ T cells was significantly higher than female CD8^+^ T cells associated with higher expression of Tc1-oriented transcription factor, T-bet, in male CD8^+^ T cells (Fig 5A, 5B and 5C); ii) the addition of an anti-IFN-γ Ab abrogated the inhibitory effect of male CD8^+^ T cells (Fig 5D); iii) rIFN-γ significantly reduced IL-4 production by male CD4^+^ T cells, but not by female CD4^+^ T cells (Fig 6); and iv) IFN-γ receptor expression was significantly higher in male CD4^+^ T cells than female CD4^+^ T cells (Fig 7A, 7B and 7C). Together with these results, higher production of IFN-γ by male CD8^+^ T cells and higher expression of IFN-γ receptors on male CD4^+^ T cells seem to induce male-dominant Th2 suppression, alternatively female-dominant Th2 differentiation, resulting in female-dominant allergic airway inflammation. The higher expression of immune regulatory molecules such as IL-10, TGF-β_1_ and perforin was observed in male than female CD8^+^ T cells (S2 Fig). The contribution of these molecules to the sex differences would need to be further investigated.

Unexpectedly, there was no sex difference in the levels of Th1 cytokines such as IL-2 and IFN-γ in the lung from WT mice (Fig 2A), even though male CD8^+^ T cells produced more IFN-γ (Fig 5A and 5B). These observations in the lung were consistent with that there were sex differences neither in the percentages of IFN-γ producing cells in CD4^+^ T cells from BLN (S1 Fig) nor in IFN-γ production by BLN cells cultured with OVA (data not shown). On the other hand, the levels of these cytokines in the lung were significantly higher (Fig 2B) and IFN-γ production by BLN cells cultured with OVA showed a tendency to be higher (data not shown) in female than male CD8KO mice. By comparison of the levels in WT mice with those in CD8KO mice in each sex, it is supposed that IFN-γ production by cells other than CD8^+^ T cells was suppressed by the presence of CD8^+^ T cells, especially in female mice. These data suggest that CD8^+^ T cells contribute to the development of the sex differences in allergic airway inflammation through the regulation of, in addition to IL-4 production by CD4^+^ T cells, Th1-related cytokine production.

Several studies have reported the suppressive activity to allergic inflammatory responses of CD8^+^ T cells is mediated by their production of IFN-γ [35–37]. However, the sex difference in IFN-γ production by CD8^+^ T cells has never been reported. Although the precise mechanisms by which male CD8^+^ T cells produce more IFN-γ were not determined in the current study, female hormone is possible to play a role in lower production of IFN-γ in female mice as reported in a previous study in which 17 β-estradiol treatment down-regulated IFN-γ expression by draining lymph node cells in the delayed-type hypersensitivity response [38].

To our knowledge, it has not been reported that IFN-γ receptor expression on CD4^+^ T cells was higher in male than female mice (Fig 7A, 7B and 7C). Several studies have reported that IFN-γ receptor expression was up-regulated by IL-1β [39], TNF-α, IL-6 [40] and prostaglandin E2 [41]. In this study, we demonstrated for the first time that IFN-γ has a potential to up-regulate the expression of IFN-γ receptor α in the both sexes (Fig 7D). Mechanisms for the up-regulation by IFN-γ remain to be determined, although IFN-γ has been reported to up-regulate the expression of receptors for IL-1β and TNF-α, which are enable to increase the receptor [39, 40]. Therefore, the sex difference in IFN-γ receptor α expression on CD4^+^ T cells might be attributable to the sex difference in synergistic and/or additive effect(s) of several types of cytokines, such as IFN-γ, IL-1β, IL-6 and TNF-α, *in vivo*. In contrast to the case of IFN-γ receptor α, IFN-γ down-regulated the expression of IFN-γ receptor β in the both sexes (Fig 7E). The down-regulation of IFN-γ receptor β expression in CD4^+^ T cells might be due to the ligand-induced auto-regulation, which is supported by Bach and coworkers’ study demonstrating ligand-induced down-regulation of IFN-γ receptor in helper T cells [42]. Therefore, one of the possible reasons for the sex difference in the expression of IFN-γ receptor β could be the difference between the sexes in mechanisms for ligand-induced down-regulation of this receptor, although the precise mechanisms were not determined in this study.

In summary, we demonstrated, as a possible mechanism for the sex differences in allergic airway inflammation, the induction of female-dominant Th2 immune responses resulting from, at least, male-dominant Th2 suppression evoked by the interaction of IFN-γ produced by CD8^+^ T cells with IFN-γ receptors expressed on CD4^+^ T cells which does not effectively work in female mice. To understand how and why sex differences in functional capacity of CD8^+^ T cells and CD4^+^ T cells are established, further studies to clarify the male-dominant Th2 suppression will be required. The present study would provide important implications for understanding the pathophysiological role of gender in asthma phenotyping, which may lead to the development of more effective management strategies for immune diseases from which mainly female patients suffer such as asthma and autoimmune diseases.

## Supporting Information

S1 Fig Sex differences in cytokine production by CD4^+^ T cells in BLN. Intracellular cytokine expression in CD4^+^ T cells was analyzed using a flow cytometer. Data are shown as the mean ± SD of four mice. Experiments were repeated twice with similar results. NS, not significant.
(TIF)

S2 Fig Gene expression of regulatory molecules in CD8^+^ T cells in BLN from male and female WT mice. The expression of *Il10*, *Tgfb1*, *Prf1* and *Fasl* in CD8^+^ T cells were measured by quantitative real-time RT-PCR. Data are shown as the mean ± SEM from at least two independent experiments (n = 7–9). *, P < 0.05; **, P < 0.01; NS, not significant.
(TIF)
