# Supplementary figures and images for: CD8+ T Cells Mediate Female-Dominant IL-4 Production and Airway Inflammation in Allergic Asthma
Source: PLoS One. 2015 Oct 21;10(10):e0140808. doi: 10.1371/journal.pone.0140808 (PMC4619475; doi:10.1371/journal.pone.0140808)

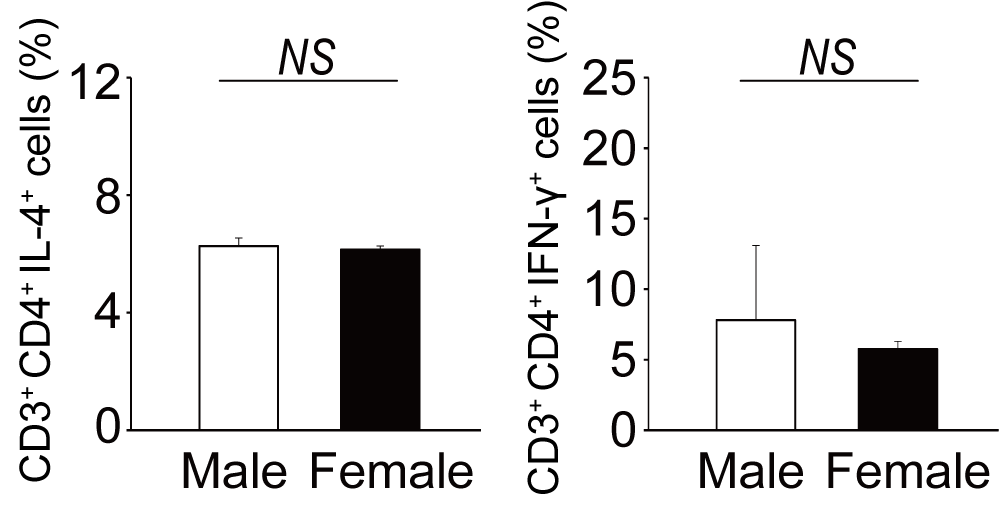

Supplement: S1 Fig — Intracellular cytokine expression in CD4+ T cells was analyzed using a flow cytometer. Data are shown as the mean ± SD of four mice. Experiments were repeated twice with similar results. NS, not significant. (TIF) [file pone.0140808.s001.tif]

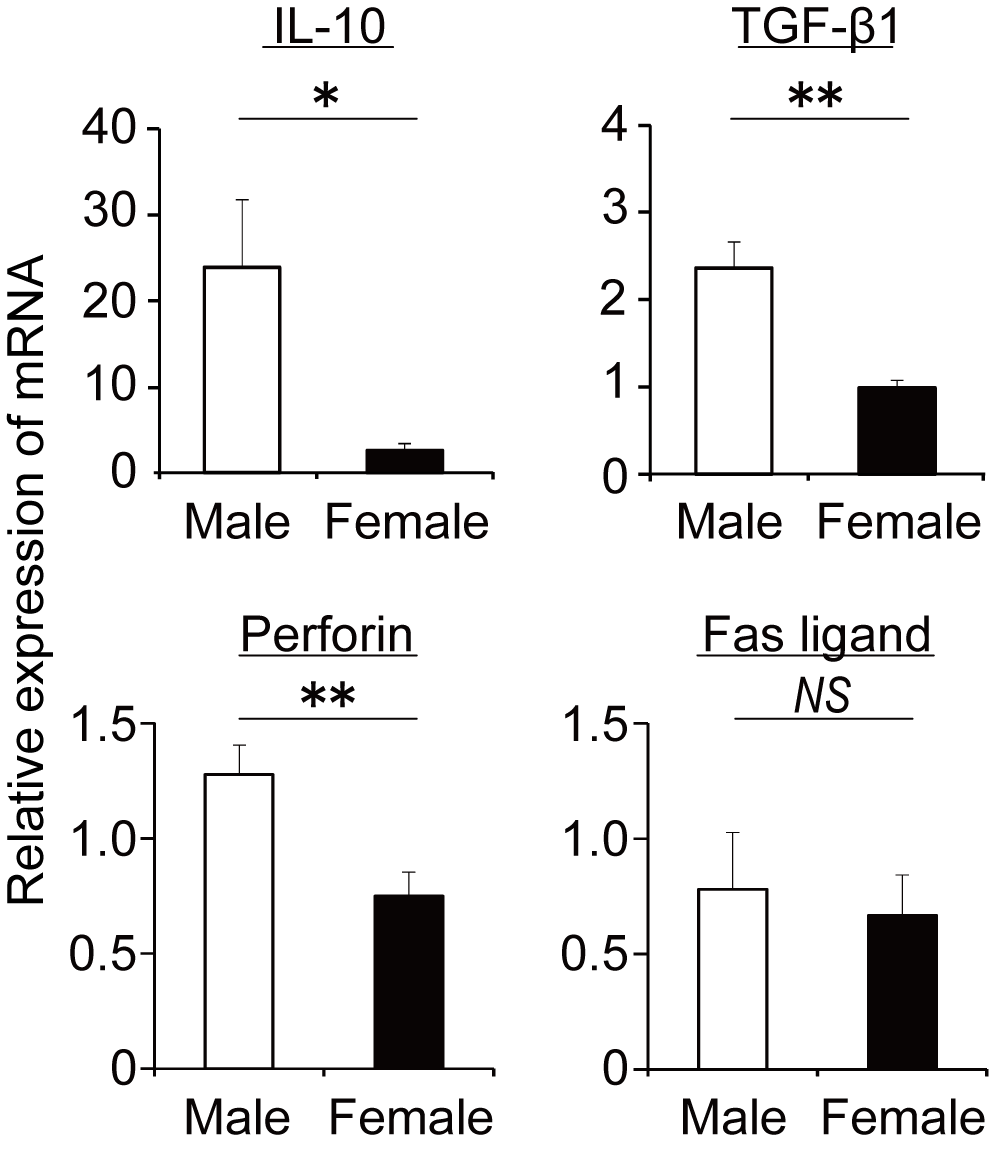

Supplement: S2 Fig — The expression of Il10, Tgfb1, Prf1 and Fasl in CD8+ T cells were measured by quantitative real-time RT-PCR. Data are shown as the mean ± SEM from at least two independent experiments (n = 7–9). *, P < 0.05; **, P < 0.01; NS, not significant. (TIF) [file pone.0140808.s002.tif]
